# Epidural analgesia for acute ischemic pain after intra‐arterial zolpidem injection in opioid‐addicted patient—A case report

**DOI:** 10.1002/ccr3.3445

**Published:** 2020-10-27

**Authors:** Irena Krajina Kmoniček, Slavica Kvolik, Kresimir Pinotić, Tomislav Ištvanić, Boris Mraovic, Ksenija Marjanovic

**Affiliations:** ^1^ Department of Anesthesiology Osijek University Hospital Osijek Croatia; ^2^ Faculty of Medicine Josip Juraj Strossmayer University of Osijek Osijek Croatia; ^3^ Department of Surgery Osijek University Hospital Osijek Croatia; ^4^ Department of Anesthesiology & Perioperative Medicine School of Medicine University of Missouri Columbia MO USA; ^5^ Department of Pathology Osijek University Hospital Osijek Croatia

**Keywords:** acute medicine, anesthesia, pharmacology, vascular surgery

## Abstract

A patient taking opioid maintenance therapy unintentionally injected dissolved zolpidem pills into the femoral artery and suffered acute limb ischemia. High amounts of opioids with supplemental therapies were inefficient for intractable ischemic pain, suggesting the presence of opioid‐induced hyperalgesia (OIH). Epidural analgesia efficiently relieved pain and symptoms of OIH.

## Graphical abstract

Intra‐arterial zolpidem injection resulted in vascular obstruction, muscle necrosis (arrowhead), acute limb ischemia, and intractable pain unresponsive to high doses of opioids. Opioid‐induced hyperalgesia (OIH) was suspected. Epidural analgesia efficiently relieved pain and symptoms of OIH in the addicted patient.

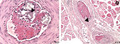

## 1 INTRODUCTION

A patient taking opioid maintenance therapy unintentionally injected dissolved zolpidem pills into the femoral artery and suffered acute limb ischemia. High amounts of opioids with supplemental therapies were inefficient for intractable ischemic pain, suggesting the presence of opioid‐induced hyperalgesia (OIH). Epidural analgesia efficiently relieved pain and symptoms of OIH.

A substantial increase in prescription opioid use was observed over the last two decades, as well as numerous patients undergoing surgery who are on maintenance therapy (OMT) with methadone or buprenorphine. These patients present a unique challenge for anesthesiologists usually requiring a complex perioperative and postoperative pain therapy.^1^, ^2^, ^3^, ^4^ Anesthesia and analgesia must be stress‐protective and sufficiently effective.^5^, ^6^ The goal of analgesia in this population is to effectively manage postoperative pain, improve patients’ satisfaction and outcomes, and reduce the cost of health care.^7^ Additionally, perioperative physicians should be aware of an impaired psychological condition, anxiety, and altered hemodynamic status in addicted patients. All these changes may be signs of withdrawal syndrome.

Opioid medications and nonsteroidal anti‐inflammatory drugs (NSAIDs) are the basic management of ischemic pain in acute limb ischemia. In addicted patients, unpredictable amounts of opioids could be required to effectively suppress ischemic pain demanding other ways for pain treatment. Challenging perioperative period is not a good time to start withdrawal therapy or to start rehabilitation of these patients.^8^ Therefore, nonopioid drugs and regional anesthesia procedures should be considered.^9^, ^10^, ^11^

We will present a perioperative approach in the treatment of acute ischemic and postoperative pain in an addicted patient who suffered acute limb ischemia with intractable ischemic pain. Despite increasing doses of opioids, his pain became worse, suggesting opioid‐induced hyperalgesia (OIH).

## 2 CASE HISTORY

A 33‐year‐old Caucasian man was admitted to a tertiary hospital in an agitated state with excruciating pain in his left leg. He was a former heroin user on OMT with methadone 45 mg PO daily. The patient unintentionally injected four dissolved zolpidem pills into his femoral artery on the day before the hospital admission. After the injection, he felt intractable pain and he took >100 mg of methadone PO with no effect.

### 2.1 Differential diagnosis and investigations

During the hospital admission in the emergency department, patient's leg was pale and cold, but with preserved peripheral arterial pulsation. He was admitted to the vascular surgery department. His initial laboratory tests suggested disseminated intravascular coagulopathy (DIC). D‐dimer was 26 400 µg/L, and platelets and fibrinogen were low. Despite peroral and parenteral rehydration, and forced diuresis with urinary alkalization, myoglobin values were still extremely high (39 159 µg/L) with a presentation of acute compartment syndrome. Color Doppler flow imaging confirmed normal blood flow at major arteries.

### 2.2 Acute pain treatment

An attending vascular surgeon has confirmed that the patient suffers acute ischemic pain that may be reduced after fasciotomy. He also suggested high doses of opioids according to the guidelines of the British National Institute for Health and Care Excellence.^12^ Since compartment syndrome was diagnosed, a fasciotomy was performed under general anesthesia with midazolam premedication, propofol, sevoflurane, and fentanyl. After fasciotomy, pain was still severe, 7‐8 on the numeric rating scale (NRS) from 0 to 10. Supplemental fentanyl 100‐200 µg IV boluses µg IV boluses were ineffective for pain relief. The postoperative course was complicated with platelet consumption and bleeding, requiring transfusions and surgical revision with hemostasis. Enoxaparin 40 mg SC twice a day was continued in the postoperative period to prevent further vascular occlusions.

A psychiatry/addiction medicine consultation suggested to continue the methadone OMT at 60 mg PO daily and diazepam 10 mg PO three times a day with meperidine IV up to 60 mg boluses. Still, the patient's pain was 9 to 10 on the NRS. He became scared and disoriented, and OIH was suspected. Diclofenac was started for his pain treatment along with diazepam to suppress anxiety, but with no changes in his status.

Due to the progression of compartment syndrome above the knee and severe pain, fasciotomy has been extended under general inhalational anesthesia 24 hours after hospital admission. Systemic opioid therapy (multiple fentanyl 100‐200 µg IV IV boluses) was continued after the surgery, and the OMT dose was increased to 100 mg PO daily. However, he was still extremely anxious, and unable to sleep and communicate. Diazepam was replaced with midazolam, and on the patient's request, diclofenac was replaced by metamizole. His pain was still unbearable. The diagnosis of flow obstruction at the minor blood vessels with severe acute ischemic pain and opioid‐induced hyperalgesia was confirmed.

On the second day after the admission (third day after zolpidem injection), he suffered painful cramps and was not able to place the affected leg into a comfort position. His plasma myoglobin levels decreased (18 829 H µg/L), diuresis was preserved, with normal urea (5.1 mmol/L) and creatinine (86 µmol/L). Platelets and packed red blood cell transfusions were given due to the ongoing bleeding with laboratory signs of DIC with platelet consumption (Hb 92 g/L, Htc 0.259, Plt 69 × 10^9^/L). The peripheral pulsations were palpable on the dorsalis pedis and posterior tibial artery, but the patient's leg was still cold with diffuse cyanotic and livid skin areas and painful cramps. His pain became intractable, both at the rest and with movement.

Epidural analgesia (EA) was offered and accepted by the patient. After platelet transfusion, a lumbar epidural catheter was uneventfully placed at the L3‐L4 levels. EA with 10 mL boluses of levobupivacaine 0.25% and fentanyl 1.2 µg/mL was started, achieving excellent pain control (NRS 0). The methadone dose was reduced to his usual maintenance dose, 45 mg/daily. He had no other complaints, was able to move his leg, and had good sleep through the night.

**FIGURE 1:**
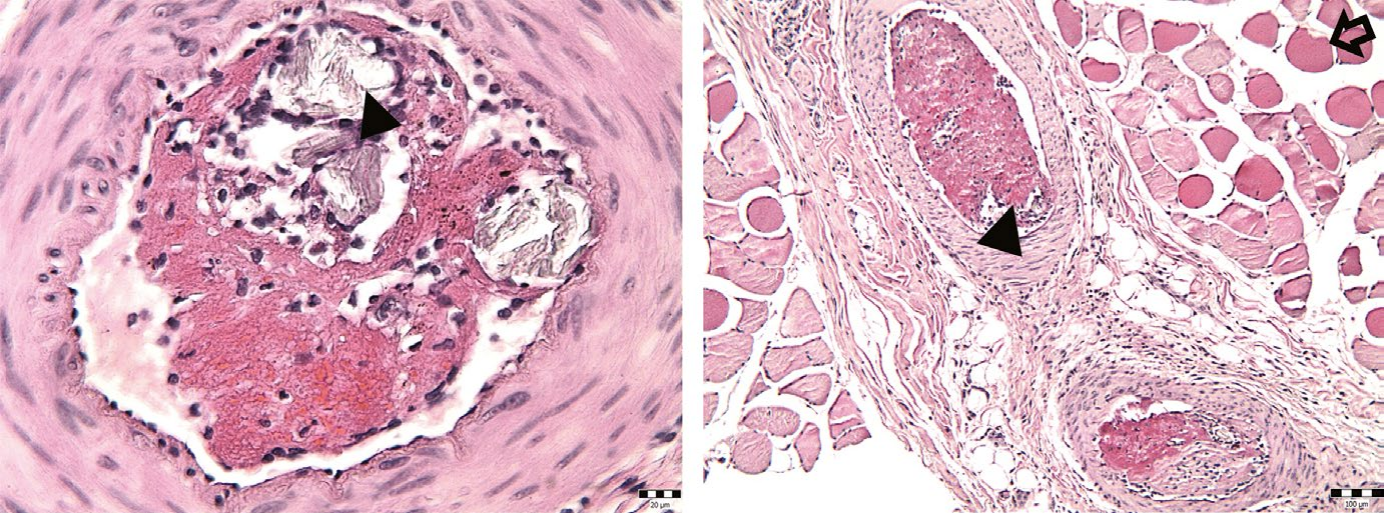
A, A thickening of vascular wall with red blood cells within, with fibrin fibers, single lymphocytes, and gray crystal‐like mass (intravascular particles of dissolved zolpidem tablet shown by arrowhead; hematoxylin and eosin stain, 400×). B, The necrotic muscle fibers surrounding blood vessels are visible and marked by open arrow (hematoxylin and eosin stain, 100×)

On the third day of hospitalization, physical examination revealed a cold left leg with multiple pale and cyanotic skin areas, suggestive for focal areas of microvascular thromboses and embolizations with skin and muscle necroses (Figure 1). Posterior tibial and dorsal pedal pulses were palpable. Diuresis was preserved with normal urea and creatinine; however, his urine became bloody. Despite all therapeutic measures, no recovery of tissue perfusion was observed. Finally, amputation above the knee was recommended by a multidisciplinary vascular team and done under EA. Postoperative EA was continued during the following 2 days. No additional systemic opioid therapy nor an increase in the dose of methadone was needed. On postoperative day three, the patient was calm and communicative. The epidural catheter was removed and methadone OMT with NSAIDs was continued. The postamputation pain reported by the patient was 2‐3 on NRS.

### 2.3 Outcome and follow‐up

The patient was discharged to home care on day 6 after a leg amputation. He had ordinary postoperative recovery with usual OMT dose and NSAIDs analgesia. After the rehabilitation, he got the prosthesis and was able to walk well.

**FIGURE 2:**
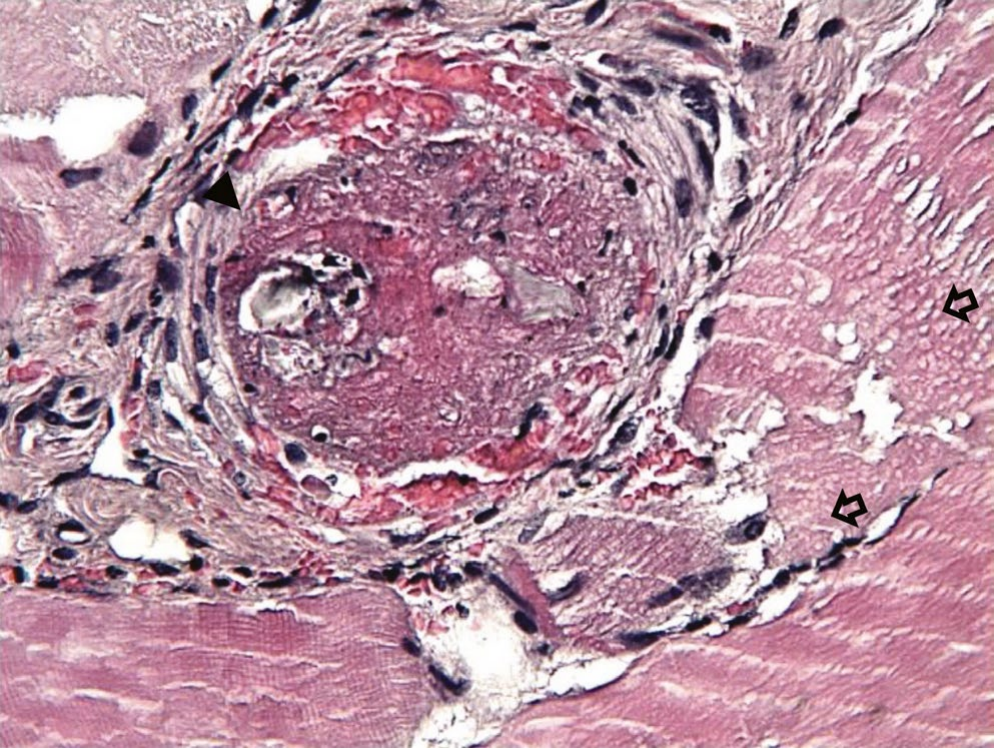
Acute ischemic lesions of muscles, including loss of cross‐striations, vacuolar degeneration typical for muscle necrosis (open arrow), and edema between myofibrils. In the left lower part of figure, there are normal striated muscle fibers (hematoxylin and eosin stain, 400×)

Histopathology specimens of his amputated leg showed multiple necrotic muscle fibers and vascular occlusions caused by the intra‐arterial injection of zolpidem. Histology slides showed severe inflammatory reactions within arteries in the areas surrounding foreign body particles (Figure 1A,B), as well as loss of cross‐striations and vacuolar degeneration in the muscles (Figure 2).

## 3 DISCUSSION

Perioperative pain control in OMT patients is often cumbersome and problematic for all parties involved patients, nurses, anesthesiologists, and surgeons. Interruption or discontinuation of OMT in patients with a history of opioid use disorder is not recommended.^13^ Anxiety, depression, delirium, lack of insight toward disease, and “coping” strategies may occur together with withdrawal syndrome and insufficient pain treatment.^14^ All these psychological problems in the perioperative period made hard to achieve effective pain control in our patient with acute limb ischemia.

Although opioids with NSAIDs are the mainstay of pain treatment in acute limb ischemia, regional anesthesia (RA) techniques are suggested as supplemental therapy if high doses of opioids are required for pain control or if the rotation of opioids is ineffective (NICE). RA provides effective pain control but may be associated with several adverse events in acute limb ischemia. It can mask acute ischemic pain and thus lead to delay of surgical or endovascular interventions. Furthermore, RA may result in hypotension, tachycardia, bradycardia, or toxic reactions, especially in patients with cardiac comorbidities.

In our patient on OMT, both increasing doses and rotation of opioids were ineffective, and the pain was getting worse with altered mental status. These clinical signs are suggestive for OIH.^16^, ^17^ Opioid‐induced hyperalgesia may result from neuroplastic changes in the peripheral and central nervous system (CNS). It may lead to pathological sensitization of pronociceptive pathways.^16^ According to the recent literature, multimodal therapy should be applied in the presence of OIH, including opioid switching, NSAIDs, and supplemental sedative medications.^17^, ^18^ After all these therapeutic options failed in our patient, we achieved good pain control using EA.

A choice of anesthetic technique should be discussed with the patient and surgeon before surgery. General anesthesia may be used as a rescue method in patients with intractable pain, but RA may be a preferable technique. It could easily achieve both adequate postoperative analgesia and peripheral vasodilatation. Suitable regional anesthesia/analgesia techniques may also decrease fear and increase patient's compliance.

However, RA has a few disadvantages in vascular patients and must be used cautiously. It could mask ischemic pain, which is an important clinical sign in the case of limb ischemia and acute compartment syndrome. Therefore, RA techniques must be followed by proper clinical, neurological, and laboratory monitoring. Prevention of an opioid withdrawal syndrome should be considered, and opioid maintenance therapy continued in the perioperative period in addicted patients undergoing RA procedures.^13^ Coagulopathy is common in acute limb ischemia. Moreover, severe inflammatory response to the foreign intravascular microparticles may promote acute vessel occlusion that requires therapeutic anticoagulation to preserve circulation. In such a complicated patient, proper balancing should be achieved between prothrombotic environments and bleeding due to coagulation factor consumption or anticoagulation therapies administered. Platelets and coagulation factor consumption must be recognized and substituted to allow a safe performance of continuous central neuraxial blocks.

## 4 CONCLUSIONS

According to the current guidelines, the use of OMT in patients with acute limb ischemia must not be discontinued in the perioperative period. The utilization of additional opioids may lead to the opioid hyperalgesia and should not be the first‐line perioperative pain treatment. Multimodal pain management strategies using regional anesthesia techniques and adjuvant opioid‐sparing techniques will allow for the best acceptable outcome.

## CONFLICT OF INTEREST

The authors declare that they have no conflicts of interest.

## AUTHOR CONTRIBUTIONS

IKK: treated the patient, analyzed literature, and drafted and reviewed the manuscript. SK: treated the patient, analyzed laboratory values, and wrote the manuscript. KM: performed the pathohistological examination and described figures. BM: revised the manuscript critically for English language and intellectual content. KP and TI: performed surgical procedures and critically analyzed manuscript, and both participated during the literature search. All authors: read and approved the final manuscript.

## INFORMED CONSENT

Since the patient was not available to give written informed consent for manuscript publication, ethics committee approval was obtained for publication of this report and accompanying images.

## Data Availability

Data sharing not applicable to this article as no datasets were generated.
